# Excision combined with ocular surface reconstruction followed by topical chemotherapy for ocular surface squamous neoplasia

**DOI:** 10.1007/s10384-024-01111-0

**Published:** 2024-10-02

**Authors:** Hiroko Nakai, Kosuke Ueda, Koji Kitazawa, Hideki Fukuoka, Tsutomu Inatomi, Norihiko Yokoi, Shigeru Kinoshita, Go Horiguchi, Satoshi Teramukai, Chie Sotozono

**Affiliations:** 1 Department of Ophthalmology, Kyoto Prefectural University of Medicine, 465 Kajii-cho, Hirokoji-agaru, Kawaramachi-dori, Kamigyo-ward, Kyoto, 602-0841 Japan; 2 Department of Ophthalmic Plastic and Orbital Surgery, Seirei Hamamatsu General Hospital, Hamamatsu City, Japan; 3 Department of Ophthalmology, National Center for Geriatrics and Gerontology, Obu City, Japan; 4 Department of Frontier Medical Science and Technology for Ophthalmology, Kyoto Prefectural University of Medicine, Kyoto, Japan; 5 Department of Biostatistics, Kyoto Prefectural University of Medicine, Kyoto, Japan

**Keywords:** Conjunctival intraepithelial neoplasia (CIN), Mitomycin-C (MMC), Ocular surface squamous neoplasia (OSSN), Squamous cell carcinoma (SCC), 5-fluorouracil (5-FU)

## Abstract

**Purpose:**

To investigate the visual prognosis of ocular surface squamous neoplasia (OSSN) after tumor resection and ocular surface reconstruction, and clarify factors that influence recurrence.

**Study design:**

Retrospective cohort study.

**Methods:**

Medical records of all patients who underwent surgical treatment for OSSN at our hospital between January 1996 and December 2019 were reviewed. Tumor size/location, histological classification, surgical procedure, intraoperative mitomycin-C use, and postoperative topical 5-fluorouracil (5-FU) administration were examined, and pre and postoperative visual acuity (VA) were compared to elucidate factors that influence disease recurrence.

**Results:**

Tumor excision was performed in 70 eyes of 70 cases (43 men, 27 women; average age: 71.6 ± 12.6 years) with dysplasia (8 eyes), carcinoma in situ (26 eyes), and invasive squamous cell carcinoma (36 eyes). Tumors were found in the limbus (*N* = 59 eyes), palpebral conjunctiva (*N* = 8 eyes), and from the bulbar to palpebral conjunctiva (*N* = 3 eyes). Surgical procedures performed were limbal transplantation/keratoepithelioplasty (*N* = 29 eyes), cultivated oral mucosal epithelial transplantation (*N* = 3 eyes), and auto-conjunctival epithelium transplantation (*N* = 2 eyes). Ocular surface was reconstructed using amniotic membrane, donor cornea, or cultivated epithelial sheet. The mean follow-up was 38.6 ± 38.6 months (range, 2 months to 13.8 years). VA postoperatively improved in 25 (61.0%) cases. Recurrence occurred in 19 (27.1%) cases at from 2 to 50 months (median: 12.5 months) postoperative. Uni- and multivariate analyses revealed that presurgical tumor size and postoperative administration of 5-FU were significantly related to recurrence.

**Conclusion:**

Combined surgical excision and postoperative topical 5-FU administration effectively prevented OSSN recurrence, and ocular surface reconstruction contributed to improvement of VA.

## Introduction

Conjunctival/corneal intraepithelial neoplasia (CIN) and invasive squamous cell carcinoma (SCC) are comparatively rare diseases with a reported incidence rate of 0.13–1.9 per 100,000 persons [1]. Although rare, they are reportedly the most common types of ocular surface squamous neoplasia (OSSN) associated with epithelial cells [2–5]. In advanced cases of OSSN that include the pupillary area, the tumor impairs best-corrected visual acuity (VA) (BCVA). Generally, the standard treatment for OSSN is surgical excision, yet the rate of recurrence post surgery is reportedly high (i.e., 21–39%) [6–8]. Thus, one primary aim of surgical treatment is to achieve an improvement of BCVA, while another is to prevent the recurrence of the disease.

In cases where the limbal epithelial defect extends to more than half of the circumference due to tumor removal, corneal epithelial transplantation (i.e., limbal transplantation and corneal epithelial plasty) is performed to provide epithelial supply and prevent conjunctival invasion (pseudopterygia) [9]. In cases in which the tumor involves less than 50% of the entire circumference of the limbus, epithelial transplantation is not always necessary. However, to prevent the occurrence of pseudopterygium, the use of a cryopreserved donor cornea is useful. In cases in which conjunctival tissue defects occur over a wide area, amniotic membrane transplantation (AMT) is used to compensate for the tissue loss and prevent adhesions and scarring. Amniotic membrane is the innermost layer of the human placenta, and it has the characteristic of promoting epithelial growth and differentiation with less fibrosis [10, 11]. AMT makes it possible to excise large tumors with a excellent margin of safety [12].

Over the past few decades, the use of various topical chemotherapeutic treatments for CIN/SCC, such as 5-fluorouracil (5-FU) [13–18], mitomycin-C (MMC) [19–26], and interferon (IFN)-α-2b [27–33] have been reported. There are some reports that the intraoperative use of MMC reduced the tumor recurrence rate [34]. In some of those previous studies it is reported that when those topical chemotherapeutics are used alone as the primary therapy, the size of the tumor diminished or even completely disappeared [14, 16, 19, 30]. Hence, the clinical use of topical chemotherapeutic agents for the treatment of OSSN has recently been increasing, often as the primary treatment without the need for surgical excision of the tumor. On the other hand, those reports cautioned that topical chemotherapeutics are most effective for CIN, and less effective for diseases that exist at a deeper level of the conjunctival and corneal tissue. Thus, we theorized that the combination of surgical excision and the topical administration of 5-FU post surgery would greatly reduce the risk of recurrence. Moreoever, although the benefits of adjuvant chemotherapy has previously been reported [8, 35, 36], there have been no reports focused on evaluating both disease recurrence and VA prognosis.

In this retrospective study, we analyzed the medical records of patients surgically treated for OSSN at the Department of Ophthalmology, Kyoto Prefectural University of Medicine Hospital, Kyoto, Japan to investigate visual prognosis post surgery and the factors that influence the rate of disease recurrence.

## Methods

This retrospective study involved 70 eyes of 70 patients surgically treated for OSSN at the Department of Ophthalmolgy of Kyoto Prefectural University of Medicine Hospital between January 1996 and December 2019. OSSN patients who did not undergo surgical treatment, or who were referred to us after undergoing surgical treatment at another hospital, were excluded from the study. The clinical records of all patients were reviewed in relation to their past clinical history, the site and extent of the tumor at the time of the initial examination, the histological classification, the surgical treatment applied (e.g., the combination of ocular surface reconstruction and intraoperative use of MMC), and the postoperative use of topically administered 5-FU or IFN. Factors that influence the rate of recurrence were analyzed, and the VA prognosis in the eyes that underwent ocular surface reconstruction was assessed. The main outcome measure was VA prognosis, and the secondary outcome was the rate of recurrence over a long-term postoperative period. This study was approved by the Ethics Review Committee of Kyoto Prefectural University of Medicine (Approval No.: ERB-C-1808), and was conducted in accordance with the tenets set forth in the Declaration of Helsinki.

### Detection of human papillomavirus (HPV) 16

We performed polymerase chain reaction (PCR) testing to amplify and detect HPV DNA in tear samples from 55 eyes of 55 patients after informed consent was obtained. In 15 cases, PCR tests were not performed because informed consent could not be obtained. Both the sample collection and the PCR tests were performed in accordance with our previously reported methods [37].

### Surgical procedure

In each patient, the primary surgical procedure was the excision of the tumor. Briefly, the tumor margin was confirmed prior to surgery via slit-lamp examination with fluorescein sodium and/or lissamine green staining. Then, a conjunctival incision was made more than 2 mm outside the tumor margin to ensure that the excision was complete, with the tumor then being separated and excised from the cornea and sclera and/or tarsal plate. The tumor on the corneal sclera was bluntly removed with a spatula, and if it adhered to the limbus and could not be removed, the superficial layer was removed with a scalpel.

Intraoperative MMC was used in combination with surgical excision, except in cases in which the tumor size was small. Briefly, a surgical sponge containing 0.04% MMC was applied on the tumor excision site for 4 min, followed by washing of the site with 300 ml of saline. In cases in which the palpebral conjunctiva was involved, cryotherapy was performed. Ocular surface reconstruction (i.e., AMT and/or epithelial transplantation) was performed depending on the site and extent of the tumor.

The tumor was submitted for pathological examination to determine the extent of the tumor. If the margin was positive on the side where margins could not be obtained, such as on the eyelid margin or corneal side, careful follow-up was conducted. Since a margin of 2 mm or more could be secured on the conjunctival side, no cases of positive margins were observed.

### Postoperative topical chemotherapy

After confirming both malignancy by pathological examination and complete epithelialization and stabilization of the ocular surface post surgery, 1% topical 5-FU was administered 4-times daily for 1 week as 1 cycle post surgery to prevent a recurrence of the tumor, with the frequency and duration of the applications being determined based on the extent of the lesions and the pathological findings. In addition, 5-FU was prescribed at the outpatient visits at 2–3 months postoperative. Moreover, topical IFN (1 M IU/ml) was administerd 4-times daily for at least 1 month.

### Statistical analysis

For statistical analysis, the number and percentage of patients were tabulated in regard to tumor size and location. For patient characteristics, we calculated the median and range for the continuous variables, and the number and percentage of cases per category for the categorical variables. A univariate and multivariate Cox proportional hazards model was used to identify the prognostic factors of recurrence. All variables, except HPV-16 with missing values, were included in the multivariate analysis. Moreover, Cox proportional hazards model was used to evaluate the effect of the treatment (i.e., surgical intervention and MMC, 5-FU, and IFN administration) on the prevention of recurrence, with patient age, sex, pathological diagnosis, lesion location, and tumor size included as covariates in the model. Kaplan–Meier survival curve analysis was used to estimate the probability of disease recurrence with or without postoperative application of topical 5-FU. In addition, scatter plots were created to examine the relationship between pre- and postoperative BCVA, and a two-sided *P* value of < 0.05 was considered statistically significant. Python version 3.8.5 (available at https://www.python.org/), lifelines version 0.26.0 (available at https://lifelines.readthedocs.io/en/latest/), and SAS version 9.4 (SAS Institute Inc. Cary, NC) statistical software was used for the analysis.

## Results

### Patients

Surgery was performed in 70 eyes of 70 cases (43 men and 27 women), and patient age at diagnosis ranged from 32 to 91 years (median age: 71.6 ± 12.6 years). The postoperative follow-up period ranged between 2 months and 13.8 years (median period: 38.6 ± 38.6 months).

### Tumor size and location

At the initial examination, the tumor was found in the limbus in 59 patients, in the palpebral conjunctiva in 8 patients, and from the bulbar to the palpebral conjunctiva in 3 patients. Among the cases in which the tumor was located on the limbus, it was found to be present within 1 quadrant in 31 patients, within 2 quadrants in 15 patients, within 3 quadrants in 8 patients, and in all quadrants in 5 patients (Table 1).

**Table 1:** Demographic characteristics, diagnoses, and treatments of the study participants

	Recurrence (+) *n*=19	Recurrence (-) *n*=51	All *n*=70
Age - year, median (range)	76 (33, 91)	78 (32, 89)	76 (32, 91)
Sex - male, n (%)	11 (57.9)	32 (62.8)	43 (61.4)
Pathological diagnosis, n (%)			
Dysplasia	1 (5.3)	7 (13.7)	8 (11.4)
CIN	7 (36.8)	19 (37.3)	26 (37.1)
SCC (TNM: T1)	0 (0)	5 (9.8)	5 (7.1)
SCC (TNM: T2)	9 (47.4)	20 (39.2)	29 (41.4)
SCC (TNM: T3)	2 (10.5)	0 (0)	2 (2.9)
Lesion location, n (%)			
Corneal limbus			
< 1 quadrant	5 (26.3)	26 (51.0)	31 (44.3)
< 2 quadrants	4 (21.1)	11 (21.6)	15 (21.4)
< 3 quadrants	3 (15.8)	5 (9.8)	8 (11.4)
All quadrants	4 (21.1)	1 (2.0)	5 (7.1)
Palpebral conjunctiva	1 (5.3)	7 (13.7)	8 (11.4)
In bulbar & palpebral conjunctiva	2 (10.5)	1 (2.0)	3 (4.3)
Size - mm, median (range)	7.0 (3.0, 17.0)	10.0 (6.0, 20.0)	8.0 (3.0, 20.0)
HPV16 - positive, n (%)	2 (11.1)	3 (8.1)	5 (9.1)
Missing	1	14	15
Operation, n (%)			
LT/KEP	10 (52.6)	12 (23.5)	22 (31.4)
COMET	1 (5.3)	0 (0)	1 (1.4)
CT	0 (0)	2 (3.9)	2 (2.9)
AMT	2 (10.5)	11 (21.6)	13 (18.6)
ET + AMT	3 (15.8)	6 (11.8)	9 (12.9)
None	3 (15.8)	20 (39.2)	23 (32.9)
MMC, n (%)	15 (78.9)	43 (84.3)	58 (82.9)
5-FU, n (%)	2 (10.5)	28 (54.9)	30 (42.9)
IFN, n (%)	2 (10.5)	3 (5.9)	5 (7.1)

CIN, carcinoma in situ; SCC, squamous cell carcinoma; HPV16, human papillomavirus 16; LT, limbal transplantation; KEP, keratoepithelioplasty; COMET, cultivated oral mucosal epithelial transplantation; CT, auto-conjunctival transplantation; AMT, amniotic membrane transplantation; ET, epithelial transplantation; None, only excision of the tumor; MMC, (intraoperative) mitomycin-C; 5-FU, (postoperative) 5-fluorouracil; IFN, (postoperative) interferon-α-2b

### Histological classification

Histopathological examination of the tumor revealed that among the 70 patients, there was dysplasia in 8, carcinoma in situ in 26, and invasive SCC in 36 (Table 1).

### Surgical treatment

**Fig. 1:**
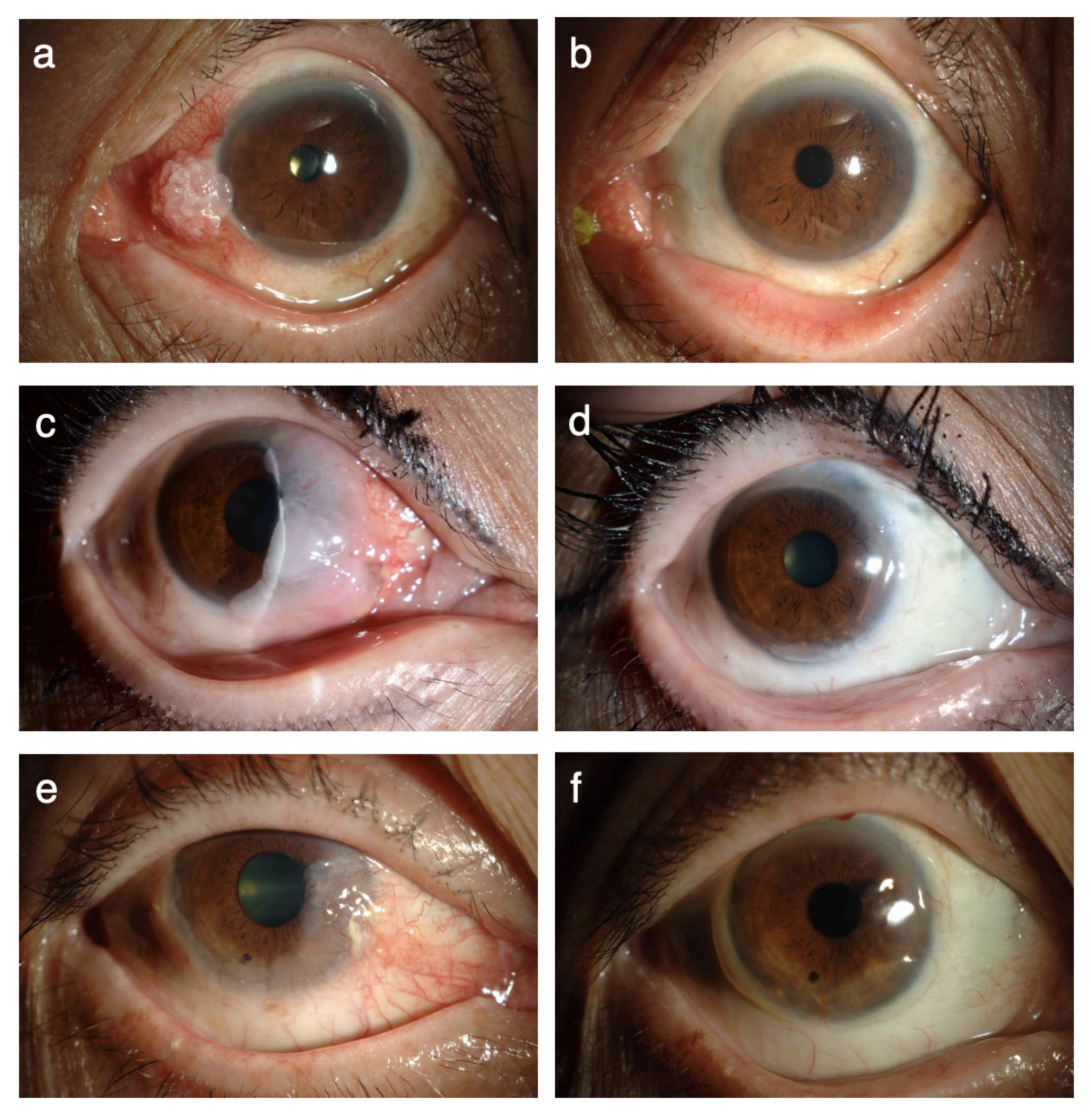
Images of ocular surface squamous neoplasia (OSSN) tumors of the corneal limbus obtained before and after epithelial transplantation. **a** Preoperative appearance of a tumor involving 1 quadrant of the corneal limbus. **b** Appearance of the eye shown in **a** after tumor excision and epithelial transplantation. **c** Preoperative appearance of a tumor involving 2 quadrants of the corneal limbus. **d** Appearance of the eye shown in **c** after tumor excision, epithelial transplantation and amniotic membrane transplantation. **e** Preoperative appearance of a tumor involving 3 quadrants of the corneal limbus. **f** Appearance of the eye shown in **e** after tumor excision and epithelial transplantation

**Fig. 2:**
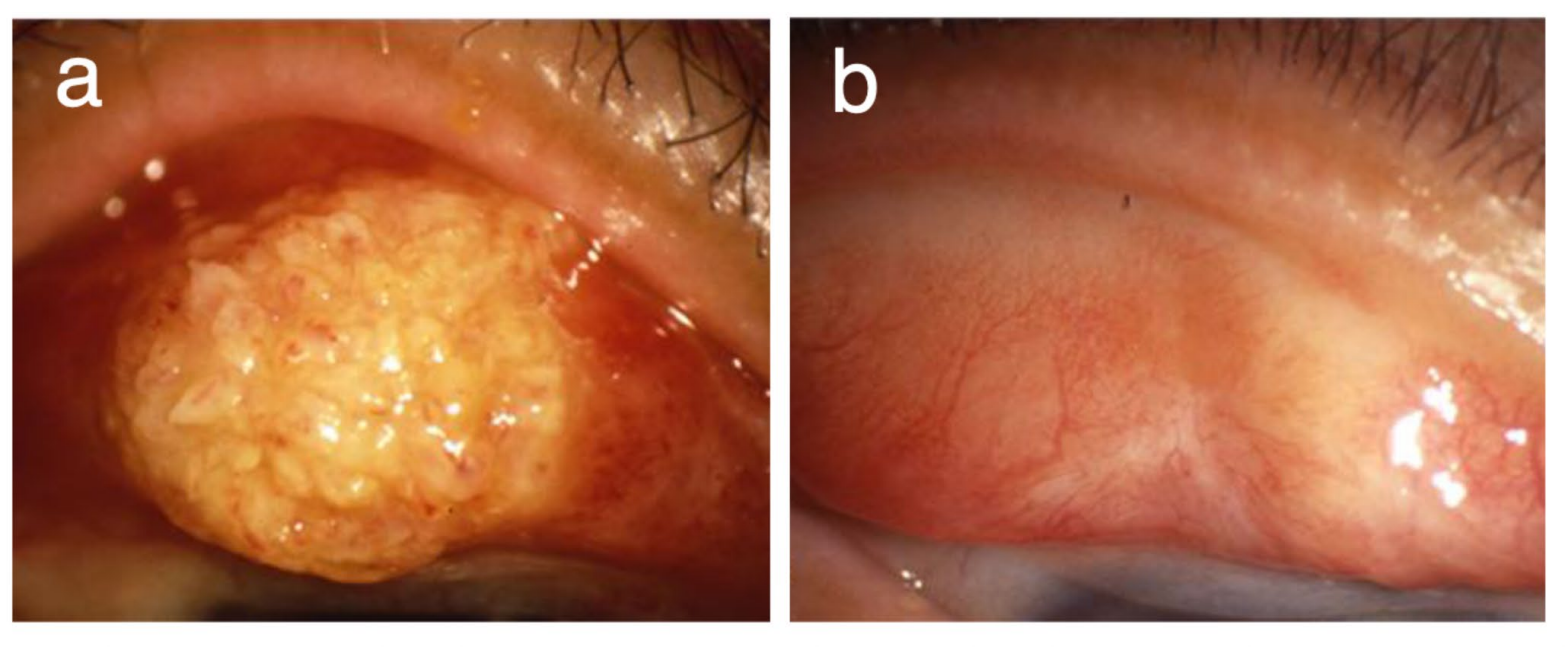
Images of OSSN tumors of the palpebral conjunctiva obtained before and after amniotic membrane transplantation (AMT). **a** Appearance of the tumor in the palpebral conjunctiva. **b** Appearance of the eye shown in **a** after tumor excision and AMT

Surgical excision of the tumor was successfully carried out in all 70 patients, and MMC was administered intraoperatively in 58 of the 70 patients. Epithelial transplantation was combined when the tumor was larger than 3 quadrants of the corneal limbus (Fig. 1). When the tumor was less than 2 quadrants of the corneal limbus, a patient-specific appropriate treatment method was selected. If the limbal epithelial defect is less than half the circumference, the epithelial supply is not needed and it is better to combine corneal transplantation to prevent pseudopterygium. In all cases, the surgeon made the final decision, taking into consideration the patient’s age, surgical time, and the availability of a donor eye. In 4 of 8 patients in which the tumor was found in the palpebral conjunctiva, and in all 3 patients in which the tumor was found from the bulbar to the palpebral conjunctiva, AMT was combined (Fig. 2; Table 2). The epithelial transplantation procedures performed in this study were limbal transplantation/keratoepithelioplasty (*N* = 29 cases), cultivated oral mucosal epithelial transplantation (*N* = 3 cases), and auto-conjunctival epithelium transplantation (*N* = 2 cases).

**Table 2:** Combination of ocular surface reconstruction

			Epithelial Transplantation								
			LT/KEP	COMET	CT	subtotal (%)		AMT (%)	ET+AMT (%)*	Total	recurrence (%)
Corneal limbus		<1 quadrant	8	0	2	10 (32.3)		5 (16.1)	2 (6.5)	31	5 (16.1)
		<2 quadrants	11	0	0	11 (73.3)		6 (40)	3 (20)	15	4 (26.7)
		<3 quadrants	7	0	0	7 (87.5)		3 (37.5)	2 (25)	8	3 (37.5)
		All quadrants	3	2	0	5 (100)		1 (20)	1 (20)	5	4 (80)
		Subtotal	29	2	2	33 (55.9)		15 (25.4)	8 (13.6)	59	16 (27.1)
Palpebral conjunctiva		palpebral only	0	0	0	0 (0)		4 (50)	0 (0)	8	1 (12.5)
		both bulbar & palpebral	0	1	0	1 (33.3)		3 (100)	1 (33.3)	3	2 (66.7)
		Subtotal	0	1	0	1 (9.1)		7 (63.6)	1 (9.1)	11	3 (27.3)
Total			29	3	2	34 (48.6)		22 (31.4)	9 (12.9)	70	19 (27.1)

ET, epithelial transplantation; LT, limbal transplantation; KEP, keratoepithelioplasty; COMET, cultivated oral mucosal epithelial transplantation; CT, auto-conjunctival transplantation; AMT, amniotic membrane transplantation

* Cases that underwent both epithelial transplantation and amniotic membrane transplantation

### Postoperative topical chemotherapy

Topical 5-FU was administered postoperatively in 30 cases (i.e., in 18 cases with invasive SCC and in 12 cases with carcinoma in situ). Topical IFN was administered postoperatively in 5 cases (i.e., in 2 cases with invasive SCC and 3 cases with carcinoma in situ).

### Visual prognosis

**Fig. 3:**
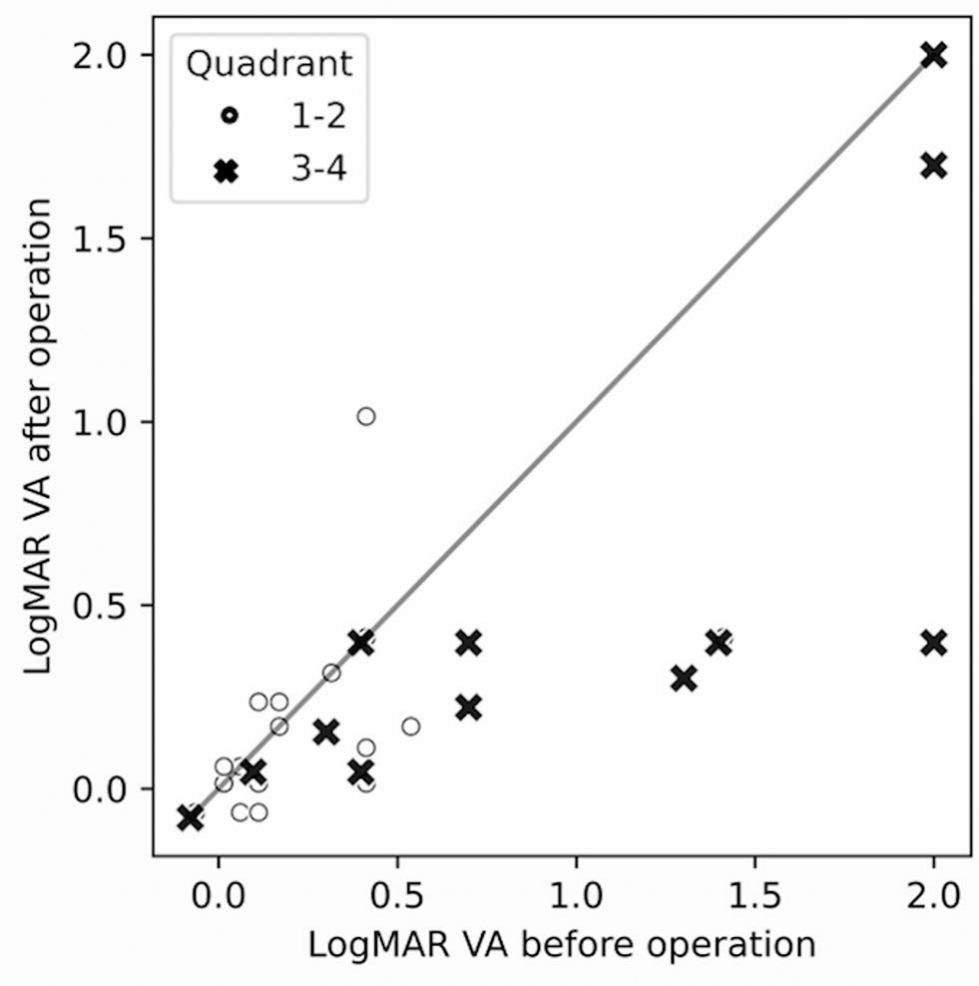
Pre- and postoperative best-corrected visual acuity of the 31 patients who underwent epithelial transplantation

When the tumor size exceded 3 quadrants, it covered the pupillary area and produced visual dysfunction. In such cases, the preoperative BCVA was less than 20/20, yet in 25 of 31 cases it improved postoperatively through the combination of epithelial transplantation (in 3 cases that underwent epithelial transplantation, the preoperative BCVA data was missing) (Fig. 3).

### Recurrence

Fluorescein staining and scleral scattering were performed at every follow-up visit in order to detect any new abnormal appearances of the epithelium, and “recurrence” was defined as the appearance of a new tumor lesion post surgery. Post surgical excision, recurrence of the tumor was clinically observed in 19 (27.1%) of the 70 patients at from 2 months to 50 months (median: 12.5 months) postoperative (Tables 1 and 2). For histological classification, the rate of recurrence was: dysplasia, 1 (12.5%) of 8 patients; SCC in situ, 7 (26.9%) of 26 patients; and invasive SCC, 11 (30.6%) of 36 patients.

The average tumor size in the patients in whom recurrence occurred was 10.5 ± 3.4 mm, while that of the patients without recurrence was 8.0 ± 3.3 mm. Recurrence occurred in 5 (16.1%) of 31 cases in which the tumor was localized to less than 1 quadrant of the limbus, in 4 (26.7%) of 15 cases in which it involved 2 quadrants, in 3 (37.5%) of 8 cases in which it involved 3 quadrants, in 4 (80.0%) of 5 cases in which it involved all quadrants, in 1 (12.5%) of 8 cases in which it involved the palpebral conjunctiva, and in 2 (66.7%) of 3 cases in which it involved both the bulbar and the palpebral conjunctiva.

Although MMC was administered intraoperatively in 58 patients, recurrence occurred in 15 (25.9%) of those patients. However, recurrence also occurred in 4 (33.3%) of the 12 patients in whom MMC was not administered intraoperatively.

**Fig. 4:**
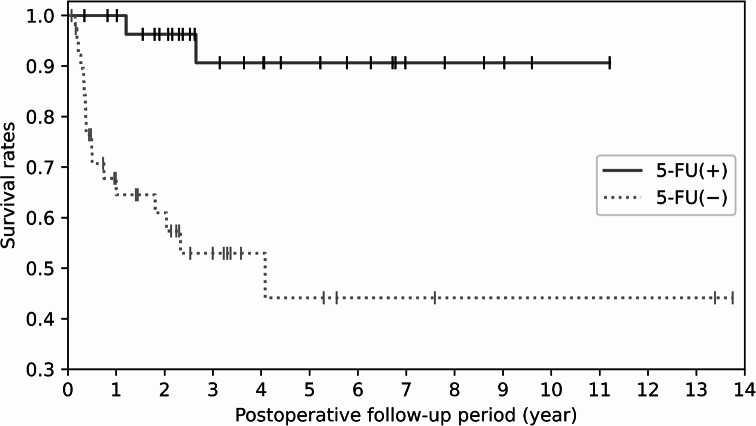
Kaplan-Meier survival curve analysis estimate of no recurrence for all patients. Results are shown for the group in which topical 5-fluorouracil (5-FU) was administered postoperatively (solid line) and the group in which topical 5-FU was not administered postoperatively (dotted line)

Among the 30 cases in which topical 5-FU was administered postoperatively (i.e., 18 cases with invasive SCC and 12 cases with carcinoma in situ), recurrence was observed in only 2 (6.7%) of those cases. Of note, patients in whom topical 5-FU was not administered post surgery had a significantly higher incidence of recurrence compared to the patients in whom it was administered (Fig. 4).

### Uni- and multivariate analyses of risk factors for OSSN recurrence

Among the prognostic factors, the rate of postoperative recurrence was found to be related to patient age (hazard ratio [HR] = 1.05, 95% confidence interval [CI] 1.00 to 1.09, *P* = 0.028), lesion location (i.e., in bulbar and palpebral conjunctiva: HR = 7.17, 95% CI 1.10 to 46.73, *P* = 0.039), and tumor size (HR = 1.20, 95% CI 1.01 to 1.41, *P* = 0.037) (Table 3). All eyes in which the tumor location was in the bulbar and palpebral conjunctiva presented an extended large size of OSSN.

**Table 3:** Uni- and multivariate analyses of risk factors for OSSN recurrence (prognostic factors)

	Univariate			Multivariate *n* = 70		
Variable	HR	95%CI	P value	HR	95%CI	P value
Age - year	1.02	[0.98, 1.06]	0.297	1.05	[1.00, 1.09]	0.028
Sex - Male	0.81	[0.32, 2.01]	0.646	0.69	[0.26, 1.81]	0.449
Pathological diagnosis						
Dysplasia	Ref.			Ref.		
CIN	2.02	[0.25, 16.45]	0.511	2.82	[0.37, 21.36]	0.316
SCC	2.46	[0.32, 19.07]	0.389	2.85	[0.41, 19.81]	0.289
Lesion location						
Corneal limbus (1–2 quadrant)	Ref.			Ref.		
Corneal limbus (3–4 quadrant)	2.79	[1.04, 7.50]	0.043	1.92	[0.60, 6.13]	0.271
Palpebral conjunctiva	0.55	[0.07, 4.35]	0.570	1.35	[0.19, 9.63]	0.766
In bulbar & palpebral conjunctiva	4.08	[0.88, 18.95]	0.073	7.17	[1.10, 46.73]	0.039
Size - mm	1.19	[1.05, 1.34]	0.005	1.20	[1.01, 1.41]	0.037
HPV16 - positive	1.26	[0.29, 5.54]	0.757			

OSSN, ocular surface squamous neoplasia; HR, hazard ratio; CI, confidence interval; CIN, carcinoma in situ; SCC, squamous cell carcinoma

The rate of postoperative recurrence was found to not be statistically related to the detection of HPV-16 in the patients’ tear samples. Among the treatment factors, including surgical intervention, postoperative 5-FU administration was the only factor found to reduce the risk of recurrence (HR = 0.12, 95% CI 0.02 to 0.60, *P* = 0.010) (Table 4). No significant difference was observed between the patients in whom MMC was, or was not, administered intraoperatively (HR = 1.16, 95% CI 0.29 to 4.59, *P* = 0.834).

**Table 4:** Uni- and multivariate analyses of risk factors for OSSN recurrence (treatment)

	Univariate			Multivariate *n* = 70		
Variable	HR	95%CI	P value	HR	95%CI	P value
Age - year	1.02	[0.98, 1.06]	0.297	1.03	[0.98, 1.09]	0.251
Sex - Male	0.81	[0.32, 2.01]	0.646	0.79	[0.29, 2.13]	0.646
Pathological diagnosis						
Dysplasia	Ref.			Ref.		
CIN	2.02	[0.25, 16.45]	0.511	2.63	[0.35, 19.88]	0.349
SCC	2.46	[0.32, 19.07]	0.389	3.42	[0.45, 26.18]	0.237
Lesion location						
Corneal limbus (1–2 quadrant)	Ref.			Ref.		
Corneal limbus (3–4 quadrant)	2.79	[1.04, 7.50]	0.043	1.15	[0.30, 4.36]	0.836
Palpebral conjunctiva	0.55	[0.07, 4.35]	0.570	1.55	[0.21, 11.77]	0.670
In bulbar & palpebral conjunctiva	4.08	[0.88, 18.95]	0.073	17.22	[1.42, 209.59]	0.026
Size - mm	1.19	[1.05, 1.34]	0.005	1.10	[0.88, 1.39]	0.403
Operation						
None	Ref.			Ref.		
LT/KEP	3.74	[1.03, 13.59]	0.045	1.45	[0.30, 7.04]	0.645
AMT	1.17	[0.19, 6.99]	0.866	0.92	[0.12, 6.82]	0.939
Others (COMET, CT, ET + AMT)	2.84	[0.63, 12.74]	0.172	0.98	[0.11, 8.74]	0.985
MMC	0.77	[0.25, 2.32]	0.639	1.16	[0.29, 4.59]	0.834
5-FU	0.10	[0.02, 0.45]	0.003	0.12	[0.02, 0.60]	0.010
IFN	1.50	[0.34, 6.52]	0.591	1.27	[0.23, 6.95]	0.784

OSSN, ocular surface squamous neoplasia; HR, hazard ratio; CI, confidence interval; None, only excision of the tumor; LT, limbal transplantation; KEP, keratoepithelioplasty; AMT, amniotic membrane transplantation; COMET, cultivated oral mucosal epithelial transplantation; CT, auto-conjunctival transplantation; ET, epithelial transplantation; MMC, mitomycin-C; 5-FU, 5-fluorouracil; IFN, interferon-α-2b

### Adverse events

In the eyes that underwent ocular surface reconstruction, no complications such as epithelial defects, graft rejection, or infections occurred. Moreover, in all cases in which MMC was administered intraoperatively, no complications occurred. Among the 30 eyes in which 1% topical 5-FU was administered, a large corneal epithelial defect with associated eye pain suddenly occurred in 1 case at 16 days of treatment, yet the defect in that eye healed within 1 week after the treatment was discontinued. In eyes that underwent topical IFN, no complications occurred.

At the final follow-up examination, 67 of the 70 cases had no serious visual disturbance, and there were no cases with scleral invasion and no cases in which metastasis developed. In all cases, the appearence of the ocular surface was favorable; i.e., no conjunctival injection and no synechia.

## Discussion

In this retrospective study, we investigated the medical records of 70 eyes of 70 patients who were surgically treated for OSSN, and our findings revealed that BCVA improved post surgery through the combination of epithelial transplantation in 25 of 31 cases. Recurrence of the tumor was clinically observed in 19 (27.1%) of the 70 patients at from 2 to 50 months post surgical excision. Among the prognostic factors, the rate of postoperative recurrence was found to be related to patient age, lesion location, and tumor size. Among the treatment factors, postoperative 5-FU administration was the only factor that was found to reduce the risk of recurrence.

Since corneal epithelial stem cells are mainly located at the limbus [38], a surgical excision of the tumor involving the corneal limbus can induce the loss of corneal epithelial stem cells, thus resulting in visual dysfunction due to corneal conjunctivalization. Thus, in patients who undergo ocular surface reconstruction, proper patient-specific planning of the surgery is key to obtaining a favorable visual prognosis.

In this present study, 47 of the 70 cases had a widely extended tumor and needed ocular surface reconstruction in combination with the tumor excision. In all eyes that underwent epithelial transplantation, the ocular surface was successfully covered by corneal epithelium and the VA prognosis was favorable. Since AMT has the potential to prevent such scarring [12, 39], it was performed in the cases with large CIN or SCC located in the palpebral or bulbar conjunctiva. All of those cases healed without scarring, and with no functional or cosmetic problems post surgery. Previous studies have reported that histopathological findings indicate the malignancy and the mitotic activity of OSSN [1, 40]. In this present study, the histopathological diagnosis was found to be unrelated to the rate of recurrence, yet the size of the tumor was found to be significantly related to recurrence. Moreover, the location of the tumor was found to influence the rate of the recurrence, possibly due to the fact that tumors located from the bulbar to the palpebral conjunctiva presented extremely larger in size than tumors located only in the palpebral conjunctiva or the corneal limbus.

As mentioned above, to guarantee complete excision of the tumors, the conjunctival incisions were made more than 2 mm outside the tumor margin. In 58 cases MMC was intraoperatively administered in combination with the surgical excision of the tumor, and recurrence occurred in 15 (25.9%) of those cases. However, our multivariate analysis revealed that the intraoperative use of MMC did not significantly reduce the rate of recurrence.

Topically administered 5-FU can be used for the purpose of (1) therapy of the recurring tumor, (2) reduction of the tumor pre excision, and (3) prevention of postoperative recurrence. The findings of previous studies have demonstrated that the application of topical 5-FU alone can completely reduce both CIN and SCC [14–16]. However, from our experience, the tumor margins of the recurrent tumor after topical 5-FU become obscure, making the diagnosis of a recurrence difficult. In contrast, the tumor margin is usually well defined in cases with primary surgery or postoperative recurrent lesions. Thus, surgical excision of the tumor combined with postoperative topical 5-FU holds promise as a method for preventing the recurrence of OSSN.

In regard to adverse events associated with the postoperative administration of topical 5-FU, the first treatment protocol presented in this study (i.e., 3 weeks of topical 1% 5-FU administration) resulted in the development of a sudden epithelial erosion in 1 case. However, no complication was experienced when the second treatment protocol was utilized (i.e., 1 week of topical 1% 5-FU administration). Hence, topical administration of 1% 5-FU can be considered safe in the clinical setting.

It should be noted that this study had several limitations. First, the determination to administer MMC intraoperatively was left to the discretion of the surgeon who performed the operation. Second, this was a non-comparative study. Moreover, since the 1% 5-FU solution used in this study is not commercially available and must be prepared in the hospital, a prospective double-arm clinical trial was deemed too difficult to conduct. Therefore, we adjusted for confounding bias as much as possible by adding covariates to the regression model, but not for unobserved confounding.

In conclusion, our findings revealed that larger-size tumors had a higher incidence of recurrence, and that the postoperative application of topical 5-FU significantly reduced the recurrence of OSSN. Moreover, ocular surface reconstruction with surgical excision of the tumor was effective for obtaining a good VA prognosis. The combination of surgical excision and ocular surface reconstruction followed by postoperative topical 5-FU administration is a remarkably effective treatment method for cases of OSSN.

## Declarations

### Additional Information

Coauthor Kosuke Ueda, MD, died April 11, 2023.

## References

[1] Lee GA, Hirst LW. Ocular surface squamous neoplasia. Surv Ophthalmol. 1995;39:429–50. 7660300 10.1016/s0039-6257(05)80054-2

[2] Shields CL, Demirci H, Karatza E, Shields JA. Clinical survey of 1643 melanocytic and nonmelanocytic conjunctival tumors. Ophthalmology. 2004;111:1747–54. 15350332 10.1016/j.ophtha.2004.02.013

[3] Giaconi JA, Karp CL. Current treatment options for conjunctival and corneal intraepithelial neoplasia. Ocul Surf. 2003;1:66–73. 17075634

[4] Shields CL, Alset AE, Boal NS, Casey MG, Knapp AN, Sugarman JA, et al. Conjunctival Tumors in 5002 cases. Comparative analysis of Benign Versus Malignant counterparts. The 2016 James D. Allen Lecture. Am J Ophthalmol. 2017;173:106–33. 27725148 10.1016/j.ajo.2016.09.034

[5] Kao AA, Galor A, Karp CL, Abdelaziz A, Feuer WJ, Dubovy SR. Clinicopathologic correlation of ocular surface squamous neoplasms at Bascom Palmer Eye Institute: 2001 to 2010. Ophthalmology. 2012;119:1773–6. 22771047 10.1016/j.ophtha.2012.02.049

[6] Tabin G, Levin S, Snibson G, Loughnan M, Taylor H. Late recurrences and the necessity for long-term follow-up in corneal and conjunctival intraepithelial neoplasia. Ophthalmology. 1997;104:485–92. 9082277 10.1016/s0161-6420(97)30287-5

[7] Erie JC, Campbell RJ, Liesegang TJ. Conjunctival and corneal intraepithelial and invasive neoplasia. Ophthalmology. 1986;93:176–83. 3951824 10.1016/s0161-6420(86)33764-3

[8] Galor A, Karp CL, Oellers P, Kao AA, Abdelaziz A, Feuer W, et al. Predictors of ocular surface squamous neoplasia recurrence after excisional surgery. Ophthalmology. 2012;119:1974–81. 22704832 10.1016/j.ophtha.2012.04.022 PMC3459154

[9] Kaliki S, Mohammad FA, Tahiliani P, Sangwan VS. Concomitant simple limbal epithelial transplantation after surgical excision of ocular surface squamous neoplasia. Am J Ophthalmol. 2017;174:68–75. 27832940 10.1016/j.ajo.2016.10.021

[10] Koizumi NJ, Inatomi TJ, Sotozono CJ, Fullwood NJ, Quantock AJ, Kinoshita S. Growth factor mRNA and protein in preserved human amniotic membrane. Curr Eye Res. 2000;20:173–7. 10694891

[11] Ueta M, Kweon M-N, Sano Y, Sotozono C, Yamada J, Koizumi N, et al. Immunosuppressive properties of human amniotic membrane for mixed lymphocyte reaction. Clin Exp Immunol. 2002;129:464–70. 12197887 10.1046/j.1365-2249.2002.01945.x PMC1906465

[12] Palamar M, Kaya E, Egrilmez S, Akalin T, Yagci A. Amniotic membrane transplantation in surgical management of ocular surface squamous neoplasias: long-term results. Eye. 2014;28:1131–5. 24993317 10.1038/eye.2014.148 PMC4166637

[13] Yeatts RP, Ford JG, Stanton CA, Reed JW. Topical 5-fluorouracil in treating epithelial neoplasia of the conjunctiva and cornea. Ophthalmology. 1995;102:1338–44. 9097771 10.1016/s0161-6420(95)30866-4

[14] Yeatts RP, Engelbrecht NE, Curry CD, Ford JG, Walter KA. 5-Fluorouracil for the treatment of intraepithelial neoplasia of the conjunctiva and cornea. Ophthalmology. 2000;107:2190–5. 11097594 10.1016/s0161-6420(00)00389-4

[15] Yamamoto N, Ohmura T, Suzuki H, Shirasawa H. Successful treatment with 5-fluorouracil of conjunctival intraepithelial neoplasia refractive to mitomycin-C. Ophthalmology. 2002;109:249–52. 11825803 10.1016/s0161-6420(01)00926-5

[16] Midena E, Angeli CD, Valenti M, de Belvis V, Boccato P. Treatment of conjunctival squamous cell carcinoma with topical 5-fluorouracil. Br J Ophthalmol. 2000;84:268–72. 10684836 10.1136/bjo.84.3.268 PMC1723406

[17] Al-Barrag A, Al-Shaer M, Al-Matary N, Al-Hamdani M. 5-Fluorouracil for the treatment of intraepithelial neoplasia and squamous cell carcinoma of the conjunctiva, and cornea. Clin Ophthalmol. 2010;4:801–8. 20689797 10.2147/opth.s9709 PMC2915867

[18] Pérez-García P, Burgos-Blasco B, Gómez-Calleja V, Vidal-Villegas B, Méndez-Fernández R, Gegúndez-Fernández JA, et al. Efficacy and safety of topical 5-fluorouracil in conjunctival intraepithelial neoplasia refractory to interferon alpha-2b. J Oncol Pharm Pract. 2023;29:975–9. 36131486 10.1177/10781552221125763

[19] Frucht-Pery J, Sugar J, Baum J, Sutphin JE, Pe’er J, Savir H, et al. Mitomycin C treatment for conjunctival—corneal intraepithelial neoplasia: a Multicenter experience. Volume 104. Ophthalmology; 1997. pp. 2085–93. 10.1016/s0161-6420(97)30055-4 9400769

[20] Wilson MW, Hungerford JL, George SM, Madreperla SA. Topical mitomycin C for the treatment of conjunctival and corneal epithelial dysplasia and neoplasia. Am J Ophthalmol. 1997;124:303–11. 9439356 10.1016/s0002-9394(14)70822-0

[21] Haas K, Ben-Dor D, Levartovsky S. Treatment of conjunctival corneal intraepithelial neoplasia with topical mitomycin C. Arch Ophthalmol. 1999;117:544–5. 10206591 10.1001/archopht.117.4.544

[22] Rozenman Y, Frucht-Pery J. Treatment of conjunctival intraepithelial neoplasia with topical drops of mitomycin C. Cornea. 2000;19:1–6. 10631999 10.1097/00003226-200001000-00001

[23] Frucht-Pery J, Rozenman Y, Pe’er J. Topical mitomycin-C for partially excised conjunctival squamous cell carcinoma. Ophthalmology. 2002;109:548–52. 11874760 10.1016/s0161-6420(01)00967-8

[24] Kemp EG, Harnett AN, Chatterjee S. Preoperative topical and intraoperative local mitomycin C adjuvant therapy in the management of ocular surface neoplasias. Br J Ophthalmol. 2002;86:31–4. 11801499 10.1136/bjo.86.1.31 PMC1770962

[25] Gupta A, Muecke J. Treatment of ocular surface squamous neoplasia with Mitomycin C. Br J Ophthalmol. 2010;94:555–8. 20447963 10.1136/bjo.2009.168294

[26] Kozma K, Dömötör ZR, Csutak A, Szabó L, Hegyi P, Erőss B, et al. Topical pharmacotherapy for ocular surface squamous neoplasia: systematic review and meta-analysis. Sci Rep. 2022;12:14221. 35987957 10.1038/s41598-022-18545-6 PMC9392743

[27] Vann RR, Karp CL. Perilesional and topical interferon alfa-2b for conjunctival and corneal neoplasia. Ophthalmology. 1999;106:91–7. 9917787 10.1016/S0161-6420(99)90009-X

[28] Karp CL, Moore JK, Rosa RH Jr. Treatment of conjunctival and corneal intraepithelial neoplasia with topical interferon alpha-2b. Ophthalmology. 2001;108:1093–8. 11382635 10.1016/s0161-6420(01)00577-2

[29] Schechter BA, Schrier A, Nagler RS, Smith EF, Velasquez GE. Regression of presumed primary conjunctival and corneal intraepithelial neoplasia with topical interferon alpha-2b. Cornea. 2002;21:6–11. 11805499 10.1097/00003226-200201000-00003

[30] Schechter BA, Koreishi AF, Karp CL, Feuer W. Long-term follow-up of conjunctival and corneal intraepithelial neoplasia treated with topical interferon alfa-2b. Ophthalmology. 2008;115:1291–6. 1296.e1. 18187195 10.1016/j.ophtha.2007.10.039

[31] Karp CL, Galor A, Chhabra S, Barnes SD, Alfonso EC. Subconjunctival/perilesional recombinant interferon α2b for ocular surface squamous neoplasia: a 10-year review. Ophthalmology. 2010;117:2241–6. 20619462 10.1016/j.ophtha.2010.03.052

[32] Shields CL, Paulose SA, Yaghy A, Dalvin LA, Constantinescu AB, Lally SE, et al. Ocular surface squamous neoplasia managed with primary Interferon α2b: a comparative analysis of 212 tumors in smokers Versus nonsmokers. Cornea. 2021;40:1387–94. 33273189 10.1097/ICO.0000000000002615

[33] Shields CL, Constantinescu AB, Paulose SA, Yaghy A, Dalvin LA, Shields JA, et al. Primary treatment of ocular surface squamous neoplasia with topical interferon alpha-2b: comparative analysis of outcomes based on original tumor configuration. Indian J Ophthalmol. 2021;69:563–7. 33595473 10.4103/ijo.IJO_1665_20 PMC7942118

[34] Siganos CS, Kozobolis VP, Christodoulakis EV. The intraoperative use of mitomycin-C in excision of ocular surface neoplasia with or without limbal autograft transplantation. Cornea. 2002;21:12–6. 11805500 10.1097/00003226-200201000-00004

[35] Gichuhi S, Macharia E, Kabiru J, Zindamoyen AM, Rono H, Ollando E, et al. Topical fluorouracil after surgery for ocular surface squamous neoplasia in Kenya: a randomised, double-blind, placebo-controlled trial. Lancet Glob Health. 2016;4:e378–85. 27198842 10.1016/S2214-109X(16)30052-3 PMC5081398

[36] Hӧllhumer R, Williams S, Michelow P. Ocular surface squamous neoplasia: management and outcomes. Eye. 2021;35:1562–73. 33564137 10.1038/s41433-021-01422-3 PMC8169825

[37] Koizumi N, Nishida K, Adachi W, Tei M, Honma Y, Dota A, et al. Detection of herpes simplex virus DNA in atypical epithelial keratitis using polymerase chain reaction. Br J Ophthalmol. 1999;83:957–60. 10413702 10.1136/bjo.83.8.957 PMC1723153

[38] Kinoshita S, Kiorpes TC, Friend J, Thoft RA. Limbal epithelium in ocular surface wound healing. Invest Ophthalmol Vis Sci. 1982;23:73–80. 7085223

[39] Espana EM, Prabhasawat P, Grueterich M, Solomon A, Tseng SCG. Amniotic membrane transplantation for reconstruction after excision of large ocular surface neoplasias. Br J Ophthalmol. 2002;86:640–5. 12034686 10.1136/bjo.86.6.640 PMC1771167

[40] Ohara M, Sotozono C, Tsuchihashi Y, Kinoshita S. Ki-67 labeling index as a marker of malignancy in ocular surface neoplasms. Jpn J Ophthalmol. 2004;48:524–9. 15592775 10.1007/s10384-004-0129-0

